# Mechanism for the photodegradation of 9,10-dibutoxyanthracene in the presence of air

**DOI:** 10.1371/journal.pone.0263526

**Published:** 2022-03-11

**Authors:** Ryotaro Seto, Arisa Sato, Keita Iuchi, Shunichi Himori, Hiroaki Gotoh

**Affiliations:** 1 Department of Chemistry and Life Science, Yokohama National University, Hodogaya-Ku, Yokohama, Japan; 2 Kawasaki Kasei Chemicals Ltd., Research & Development Center, Kawasaki-Ku, Kawasaki, Japan; Nazarbayev University, KAZAKHSTAN

## Abstract

The photoreactivity of anthracene has been previously verified for a range of its derivatives. 9,10-Dibutoxyanthracene is commonly used as an electron transfer sensitizer for photopolymerization because of its favorable optical properties. This study experimentally demonstrated that 9,10-dibutoxyanthracene produces an endoperoxide species upon reaction with the oxygen present in air. A secondary decomposition product formed during the photodecomposition of the endoperoxide species was also isolated and identified. The proposed reaction pathway is supported by singlet oxygen scavenger studies and calculations of the singlet–triplet transition energies. Our findings suggest that 9,10-dibutoxyanthracene can be used as a photo-induced oxygen scavenger.

## Introduction

Anthracene consists of a conjugated skeleton that absorbs UV light at a wavelengths of ≥300 nm, and it is known that the properties of 9-monosubstituted and 9,10-disubstituted anthracenes differ greatly depending on the substituents present. For example, the fluorescence spectra, quantum yields, and UV spectra of 9,10-diarylanthracenes depend on the aryl substituent, while the reactivity of the aromatic ring also depends on the substituents [1]. In addition, some aromatic hydrocarbon compounds such as anthracene are oxidized by singlet oxygen to produce endoperoxides (EPOs), and the type of substituent, or the presence or absence of a substituent, is known to influence the reactivity [2,3]. In this context, Lemp et al. demonstrated that the monosubstitution of anthracene with an electron-donating group at the 9-position results in a solvent-dependent reactivity [3].

As another example, 9,10-dibutoxyanthracene (DBA, **1**) characteristically absorbs light in the region of 360–400 nm, and so it has potential for use as a UVA absorber (315–400 nm), which is the wavelength of light that causes skin aging. In addition, the absorption range of **1** matches the wavelength of near-UV light-emitting diodes (LEDs), which is advantageous for photoreactions, because the presence of an appropriate polymerization initiator causes photoexcited electron transfer via exciplex formation [4,5]. Compound **1** is also promising for use in fine pattern formation, as a dry film photoresist agent, and in UV curable graphic inks [6,7]. However, **1** has been found to decompose upon long-term irradiation during industrial use. Moreover, the photodegradation mechanism of **1** is yet to be elucidated.

To date, the photochemistry of anthracene and its monosubstituted derivatives has been widely studied, and the [4 + 4] photodimerization and formation of Dewar anthracene by cross-linking of the central ring have been well established [8,9]. In the case of disubstituted anthracene derivatives bearing bulky substituents at the 9- and 10-positions, such as **1**, photodimerization is unlikely because of steric repulsion at the cross-linking point [8]. Dewar anthracene, on the other hand, is thermally unstable and the reverse transformation takes place easily [9].

In this study, to investigate the possible reaction pathways involved in the degradation of **1**, this compound **1** is isolated and purified to identify its degradation products. The mechanism of the decomposition reaction is then proposed based on certain conditions (air (~78% N_2_, ~21% O_2_) or its exclusion) and by following the product and additive evolution during this process. Overall, the purpose of this study is to isolate and identify the photodegradation products, investigate the reaction pathways involved in the decomposition of **1**, and propose decomposition control methods. Although many mechanisms for generating and capturing singlet oxygen by anthracene derivatives have been reported [10], to the best of our knowledge, this paper is the first to report the formation and decomposition of EPO under the same reaction systems and light irradiation conditions.

## Materials and methods

### Materials

9,10-Dibutoxyanthracene (**1**, DBA, Kawasaki Kasei Chemical, Japan), dimethyl terephthalate (DMT, Kanto chemical, Japan), Eosin Y (EY, Fujifilm Wako Pure Chemical, Japan), furfuryl alcohol (FFA, Tokyo Chemical Industry, Japan), *N*,*N*-triethylenediamine (TEDA), 2,2,6,6-tetramethylpiperidine (TEMP), 2,6-*tert*-butyl-*p*-cresol (BHT, Tokyo Chemical Industry), *N*,*N*-dimethylformamide (DMF, Fujifilm Wako Pure Chemical), acetonitrile (ACN, Fujifilm Wako Pure Chemical), ethyl acetate (EtOAc, Fujifilm Wako Pure Chemical), and *N*-methylpyrrolidone (NMP, Tokyo Chemical Industry) were used as received without further purification.

### Equipment

TLC was performed on a plate coated with Wakogel B-5F (manufactured by Fujifilm Wako Pure Chemical, Japan) followed by drying. The UV-vis absorption spectrum of each sample was recorded using a JASCO V-550 spectrophotometer, and the NMR spectrum of each sample was recorded using a Delta ECA-500 NMR spectrometer (JASCO Corporation, Japan). Resistance to light was investigated using a SUNTEST CPS + instrument (manufactured by Taiyo Seiki Co., Ltd., 550 W/cm^2^, 300–800 nm, Japan). High-performance liquid chromatography (HPLC) was performed using an SPD-20A UV-Vis detector, a CTO-20A column oven, DGU-20A degasser, and an LC-20AD pump (all manufactured by Shimadzu Corporation, Japan).

Peaks consistent with anthracene were observed near 385 and 405 nm for **1**. In this study, a 385 nm LED light (122 mW/cm^2^, L-STND, manufactured by OptoCode, Japan) and a 405 nm LED light (133 mW/cm^2^, L-STND, manufactured by OptoCode, Japan) were used as light sources. EY has an absorption band centered at 530 nm. A 530 nm LED light (42 mW/cm^2^, L-STND, manufactured by OptoCode, Japan) was used for the reactions involving EY.

### Methods

A solution of **1** (35 mg in 3.0 mL EtOAc) was placed in a spectroscopic cell and irradiated with 405 nm light for 0, 2, 8, 16, or 20 h at room temperature (20–25°C). A quartz cell with a lid was used to prevent the entry of air (~21% O_2_) into the cell for the anaerobic experiments, and a quartz cell without a lid was used for experiments performed in the presence of air. The NMR yield of **1** in each sample was determined using ^1^H NMR spectroscopy.

FFA, TEMP, TEDA, and BHT were used as singlet oxygen scavengers. Each singlet oxygen scavenger was added at 5 wt.% (with respect to the NMP solvent) to a 1 wt.% solution of **1**, and then transferred to an SUS cup and subjected to a light resistance test in the presence of air. The residual amount of **1** was determined every hour by removing an aliquot and measuring the absorption peak at 250 nm by HPLC.

To isolate and identify the decomposition products, **1** (99.6 mg) was dissolved in DMF (3.7 mL) in a quartz cell for spectroscopic analysis, and then irradiated with 385 nm light at room temperature in presence of air for 1 or 20 h with stirring. The crude product was extracted using EtOAc, washed with saturated brine, and concentrated. TLC was performed to confirm the presence of decomposition products.

9,10-Dibutoxyanthracene-endoperoxide (DBA-EPO, **2**) was synthesized for comparison with the resulting degradation products. Compound **1** produces **2** after the addition of a photosensitized singlet oxygen generator and irradiation with an appropriate wavelength of light. In this study, EY was used as the singlet oxygen-producing dye [11]. Thus, a mixture of **1** (99.6 mg) and EY (7.1 mg) was dissolved in DMF (10 mL), and irradiated with 530 nm light in presence of air at room temperature for 4 h with stirring. The crude product was extracted with hexane, washed with saturated brine, and concentrated to obtain **2** as an almost pure compound without further purification (98.3 mg, 99% yield, as analyzed by NMR spectroscopy).

An internal standard (DMT, 11.5 mg) and **1** (15 mg) were added to a quartz cell, dissolved in DMF-*d*_7_ (1.5 mL), and irradiated with light at 385 nm in presence of air at room temperature for 20 h with stirring. Aliquots were removed during light irradiation, and the NMR yields of **1** and the degradation products were monitored over time using ^1^H NMR spectroscopy. In addition, a sample under the same conditions was irradiated with light for 1 h and then stored in the absence of light at room temperature for 19 h prior to NMR analysis. The NMR ratio of this sample was compared with that obtained after 1 h irradiation.

To investigate the factors influencing the reaction, the resolution of **1** using different solvents was investigated. Thus, compound **1** was dissolved in methanol, ethanol, acetone, acetonitrile, cyclohexane, 2-propanol, 1,4-dioxane, n-heptane, or toluene, and then irradiated with light at 385 nm in a quartz cell. The initial concentration of **1** was adjusted to approximately 1.2 × 10^−4^ M. The UV spectrum was measured during irradiation, and the residual ratio of **1** was calculated from the reduced absorbance of the peak.

Energy calculations using computational chemistry were performed to support the reaction mechanism. In all calculations, the conformation was investigated using molecular mechanics (MM3) [12], and structural optimization and vibrational analyses were performed using density functional theory (DFT) analysis at the uB3LYP/6-31+G** level of theory. Structural optimization and vibrational analysis of the singlet excited state of **1** were performed using time-dependent DFT (TD-DFT) calculation at the uB3LYP/6-31+G** level of theory. The structural optimization and vibrational analysis of **2** were performed using the uB3LYP/6-31+G** level of theory and the O-O bond dissociation enthalpies were calculated [13]. The calculation results of neutral and radical molecules of compound 2 were described in supporting infomation. The equilibrium structure was confirmed by the absence of imaginary numbers in the vibration calculations after optimization.

Medit (2000) was used to investigate the conformations [14] and Gaussian 16 [15] was used for structural optimization and vibrational analyses.

## Results and discussion

### Suppression of DBA decomposition in the absence of air

Irradiation in the anaerobic results in suppression of the photolysis of **1** compared with conditions involving atmospheric exposure. Under aerobic conditions, **1** disappeared after 2 h of irradiation. Conversely, under anaerobic conditions, approximately 90% of **1** remained after 2 h, with nearly no change being observed despite light irradiation being conducted. This loss of 10% of **1** is likely due to a stoichiometric reaction with oxygen molecules present in the system, whereby 10% of **1** was consumed immediately after irradiation.

**Fig 1:**
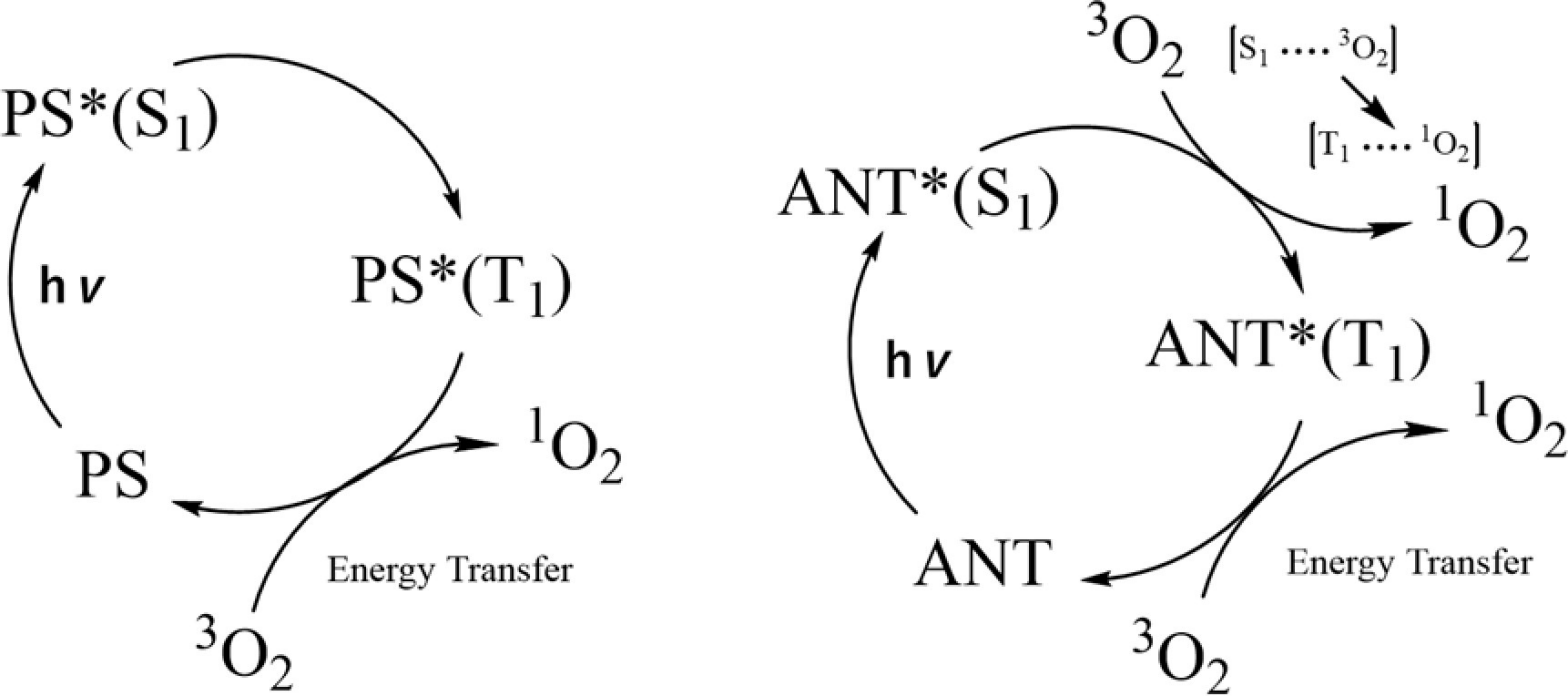
Singlet oxygen generation by anthracene derivatives from the triplet excited state of the photosensitizer (left) and singlet excited states of the anthracene derivatives (right).

Previously, anthracene derivatives have been reported to act as functional singlet oxygen generators in the photoexcited state. Many singlet oxygen generators, such as methylene blue and rose bengal, cause intersystem crossing from singlet excited states. Those in the triplet excited state produce singlet oxygen by energy sensitization with ground state oxygen. In contrast, some compounds, including anthracene derivatives, change to a singlet state, thereby forming an exciplex with ground state oxygen to produce singlet oxygen (Fig 1) [16,17]. Wilkinson et al. investigated the mechanism of singlet oxygen production from anthracene derivatives, revealing that the change in the singlet oxygen production efficiency depended on the substituents at positions 9 and 10 [16]. Anthracene derivatives can also combine with singlet oxygen to form an EPO, which has been investigated both theoretically and experimentally [18,19].

**Fig 2:**
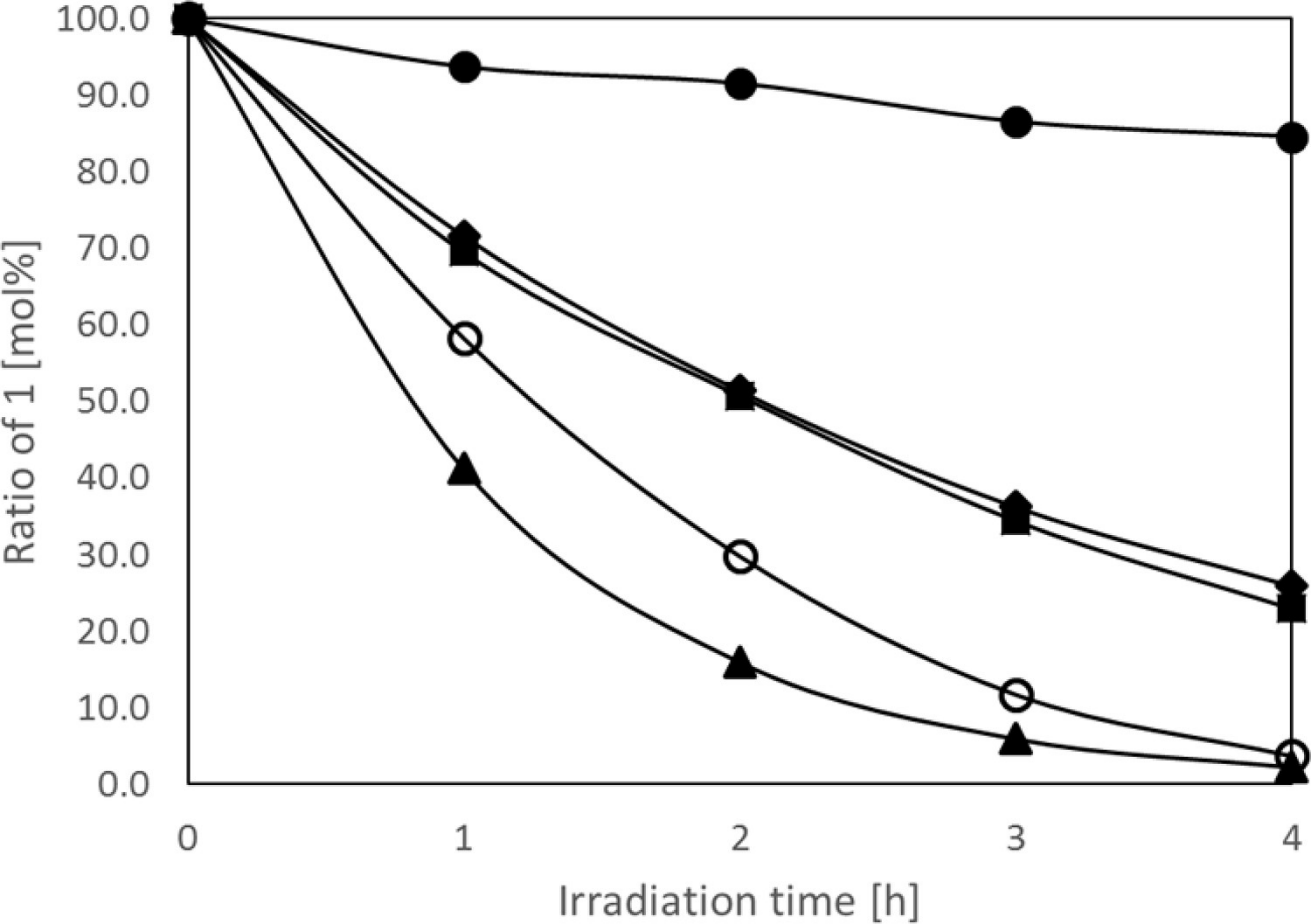
**Light resistance of 1 as a function of the light irradiation time.** The residual amount of **1** determined by HPLC (▲: blank, ◯: TEMP, ■: FFA, ◆: BHT, ●: TEDA). The concentration of all additives was 5 wt.% with respect to the NMP concentration. The wavelength of light used for irradiation was 300–800 nm (550 mW/cm^2^).

Since photodecomposition proceeds in the presence of air, it was suggested that **1** is consumed for the formation of EPO, thereby indicating that compound **1** likely plays two roles, namely the generation and capture of singlet oxygen. However, the photodecomposition of **1** was suppressed in air in the presence of additives (Fig 2), including FFA [20], TEDA, BHT [21], and TEMP [16], which are all compounds used to detect and capture singlet oxygen. Decomposition was suppressed by the addition of these compounds, thereby indicating the involvement of singlet oxygen in the photodecomposition of **1**. In particular, when TEDA was added, ~90% of **1** remained. TEDA catalytically traps singlet oxygen, while FFA, BHT, and TEMP combine with singlet oxygen and are converted to more stable compounds [20–22]. Since the relative concentrations of FFA, BHT, and TEMP added to **1** decrease as singlet oxygen capture progresses, the consumption of **1** is not suppressed.

**Fig 3:**
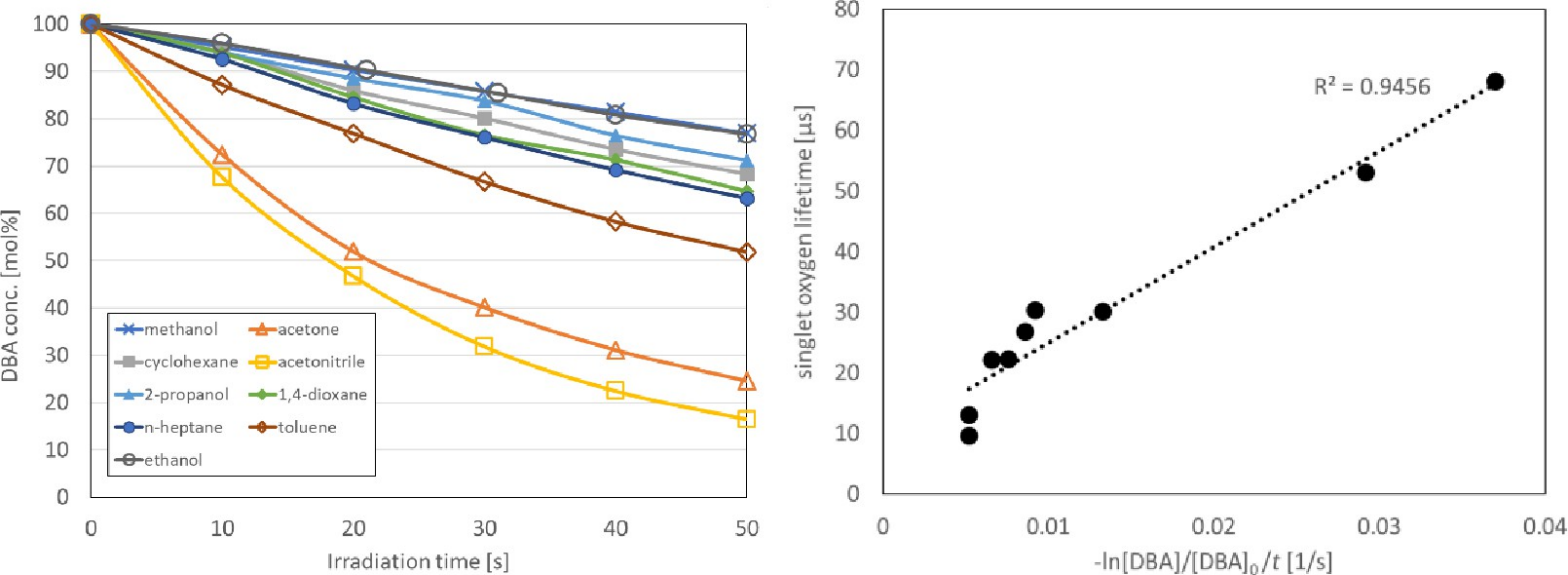
Residual amounts of **1** estimated by UV absorption in the various solvents as a function of the irradiation time (left), and decomposition rates of **1** related to the singlet oxygen lifetimes for the various solvents identified in Table 1 (right).

**Fig 4:**
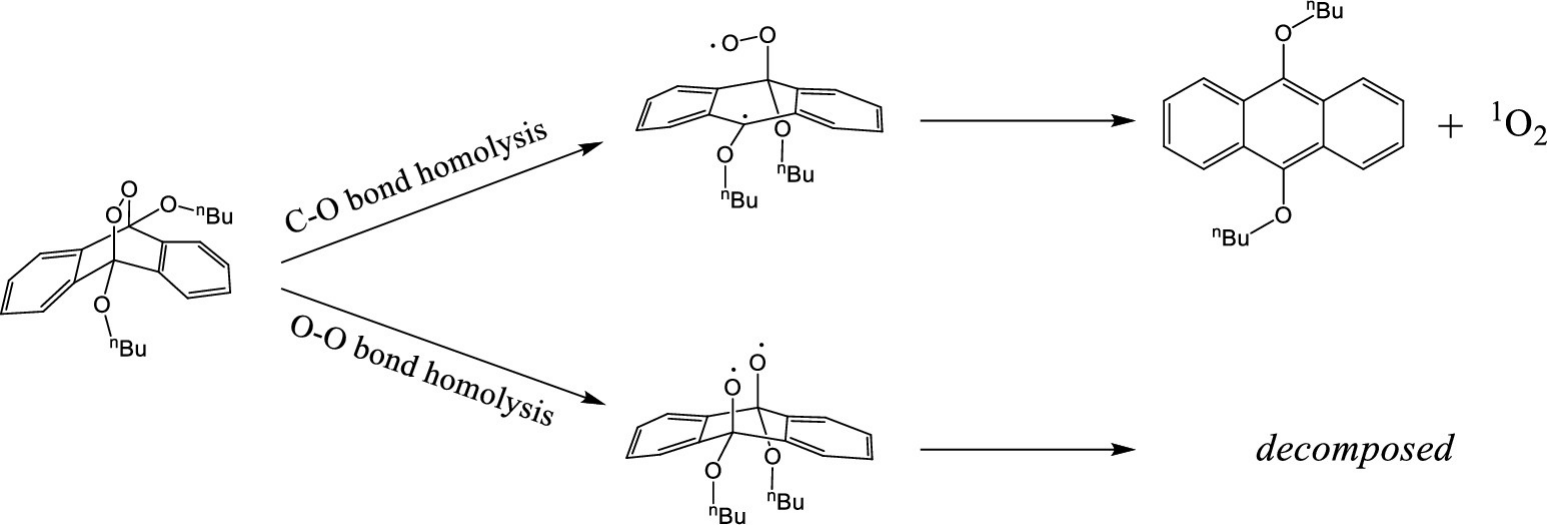
Photolysis of 1 upon capturing generated singlet oxygen and subsequent secondary decomposition to 2 and 3. Stage (ii) is proposed as the rate-determining step in the decomposition of **1** to **2**.

The decomposition rate also changed significantly upon irradiation with light in different solvents (Table 1, Fig 3A). Characteristically, the decomposition of **1** was significantly suppressed in an alcoholic solution (e.g., methanol and ethanol). In contrast, acetone and acetonitrile, despite being polar solvents, promoted the decomposition of **1**. These results indicated that there was no correlation between the rate of decomposition of **1** and the solvent polarity. However, there was a high correlation between the literature value of the singlet oxygen lifetime [23] and the decomposition rate of **1** for each solvent (Fig 3B). In solvent environments where singlet oxygen can survive for a long time, the photolysis rate of **1** was higher. This demonstrates that singlet oxygen was generated in the system and that it underwent reaction with **1**. Furthermore, this implies that the reaction between singlet oxygen and **1** is the rate-determining step (Figs 2–4).

**Table 1:** Decomposition rates of compound 1 in various solvents (−ln([DBA]/[DBA]_0_)/t) and the corresponding singlet oxygen lifetimes.

Solvent	−ln([DBA]/[DBA]_0_)/*t* [10^−3^/s]	Singlet oxygen lifetime ^[a]^ [μs]
Methanol	5.2	9.5
Acetone	29.2	53
Cyclohexane	7.6	22.2
Acetonitrile	37	68
2-Propanol	6.6	22.1
1,4-Dioxane	8.6	26.7
*n*-Heptane	9.2	30.3
Toluene	13.3	30
Ethanol	5.2	13

### Photolysis intermediates and secondary decomposition

**Fig 5:**
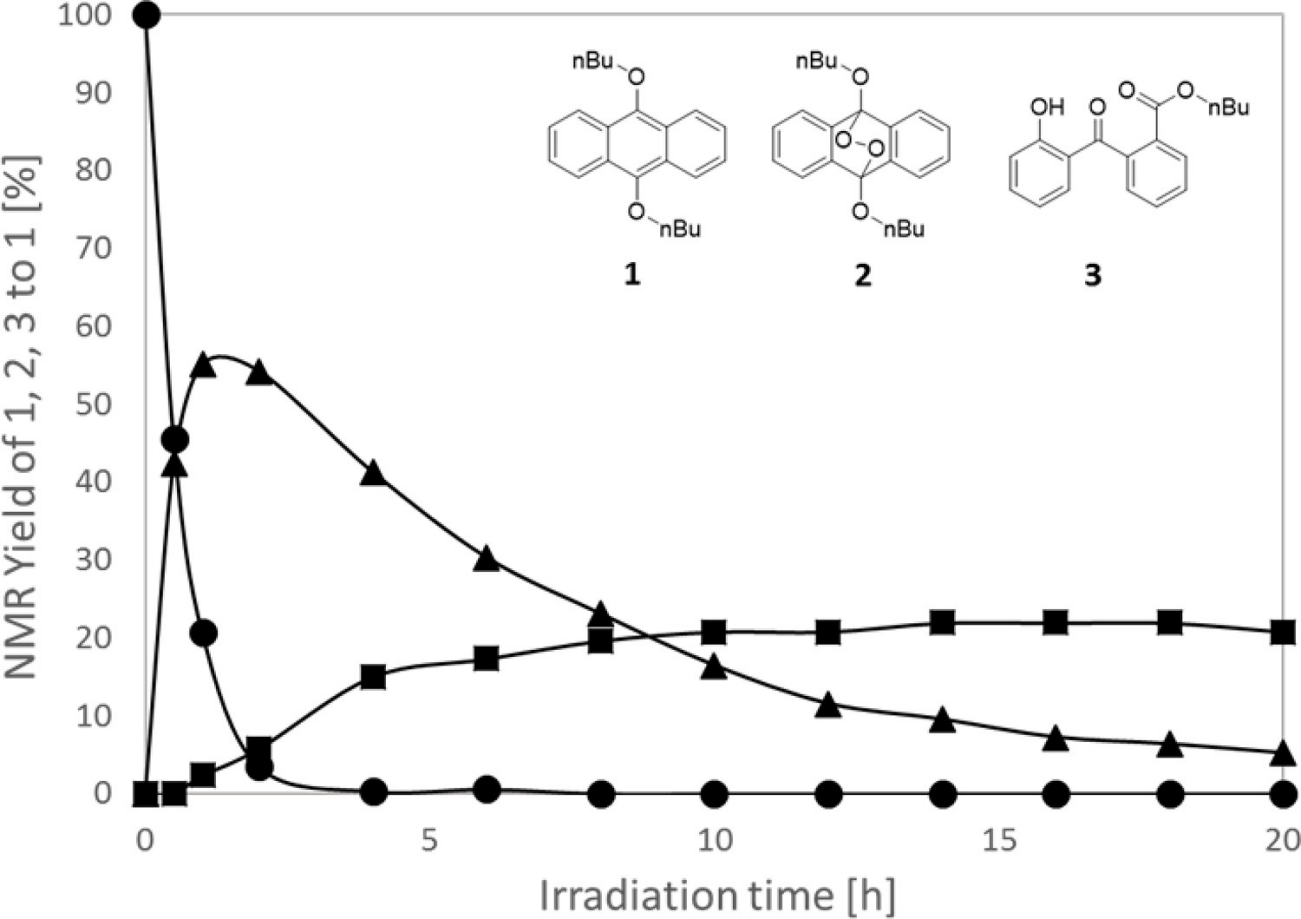
Variation in the relative NMR yields of 1, 2, and 3 (●: 1, ▲: 2, ■: 3) over time. Irradiation at 385 nm (122 mW/cm^2^). The concentration of **1** at 0 h was set to 100%.

The reaction of **1** was performed according to the same method that was used for anthracene-endoperoxide formation in a previous study [3]. In this reaction, 9,10-dibutoxyanthracene-endoperoxide (DBA-EPO, **2**) was obtained, as identified using ^1^H NMR spectroscopy and the peak shifts corresponding to EPO. The ^1^H NMR peaks of the decomposition product generated by irradiating **1** with 385 nm light for 1 h were consistent with those of **2**, while after 20 h of light irradiation, **2** disappeared, and multiple compounds were generated. Among these, **3** was identified and isolated for the first time as a photodegradation product (Fig 5). Several other compounds were observed by TLC; however, they were not characterized due to their occurrence in trace amounts or difficulties in their isolation. It was therefore considered that compound **2** produced during the initial stage of the reaction is a decomposed secondary product. Indeed, a decomposition product with a structure similar to **3**, which was obtained via the reaction between 9,10-dimethoxyanthracene-endoperoxide (DMA-EPO) and an acid, was previously isolated [24].

Thus, upon the irradiation of compound **1** with 385 nm light, the NMR yields of the reactants and products were determined over time (Fig 5). Early in the reaction, **1** disappeared and **2** appeared (~55%), and this was followed by the sequential decomposition of **2** and the generation of 3 (~20%), thereby confirming that the decomposition of **1** occurred via **2**. Compound **3** was also generated in a similar manner by irradiating **2** with EY under 385 nm light. It is likely that **1** remains in the system in small amounts because its polarity is similar to that of **2** and due to the fact that it is difficult to isolate. Since **2** absorbs very little light at 385 nm, trace amounts of **1** may absorb light and transfer energy to **2**.

Many researchers have investigated the decomposition of anthracene-endoperoxide, and in the case of thermal decomposition, the reaction has been found to differ depending on the substituents present at the 9,10-positions [24–26]. In this context, there are two potential pathways to consider, wherein one is initiated by cleavage of the C-O bond, and the other is initiated by cleavage of the O-O bond (Fig 4). The former is the reverse of the EPO production reaction that releases singlet oxygen. Thus, the difference in reactivity can be attributed to the stabilization of the biradical intermediate by the 9-position side-chain.

Anthracene-endoperoxide derivatives have also been reported to cause photodegradation [27,28], wherein C-O bond cleavage predominates in anthracene derivatives with substituents that facilitate unpaired electron delocalization, such as diphenylanthracene-endoperoxide (DPA-EPO). In this context, Drews et al. demonstrated that DPA-EPO reacts differently at two different wavelengths of light [27], resulting in cleavage of the C-O bond at one wavelength and that of the O-O bond at another.

In the case of **2**, which was obtained by the photodegradation process employed herein, cleavage of both the C-O and C-C bonds can occur. In addition, Bauch et al. reported that DMA-EPO, which has a structure similar to **2**, is decomposed by heat, acid, or base [24], and that its reaction can be initiated by the cleavage of both O-O and C-O bonds.

**Fig 6:**
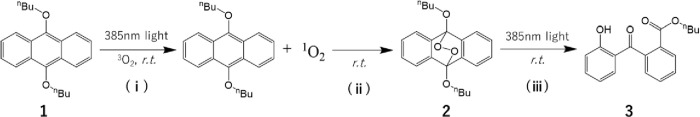
Proposed decomposition pathways for the anthracene-endoperoxide. C-O homolysis produces singlet oxygen and **1**. O-O homolysis produces degradation products via biradical intermediates.

To further investigate the decomposition conditions of **2**, it was produced from **1** using EY and isolated, followed by dissolution in N,N-dimethylformamide (DMF). It was then heated at 50°C for 20 h. However, no decomposition of **2** was observed. Therefore, it is unlikely that the decomposition of DBA-EPO is caused by heating during irradiation. The produced and isolated **2** was dissolved in DMF and irradiated with 385-nm light for 38 h, affording a crude product containing 3 (54% NMR yield, Fig 6). Heating **2** at 140°C for 14 h in a similar system afforded a crude product containing 47% of **3** and 12% of **1**. Therefore, **3** was produced predominantly by both optical and thermal decomposition. It is possible that cleavage of the C-O bond proceeds simultaneously. However, since C-O cleavage leads to a reversible reaction that produces 1 and oxygen molecules, it is highly likely that the final product will not be affected if O-O bond cleavage proceeds at simultaneously. Since 1 was not detected as the final product, the effect of C-O bond cleavage as a side reaction is small. Furthermore, **2** did not decompose at room temperature (20–25°C) or in the dark. Compared with substitution of aryl groups at the 9- and 10-positions of anthracene, the butoxy groups of 2 are less likely to delocalize the radical electrons of the reaction intermediate generated by the cleavage of the C-O bond. Therefore, it is presumed that cleavage of the O-O bond also occurs. At high temperature, a similar pyrolysis pathway under DMA-EPO conditions was also identified [24].

Based on the above results, it was confirmed that the photodecomposition of **1** is due to the production of **2** via singlet oxygen generation and capture (Fig 5). Furthermore, we found that **2** undergoes secondary decomposition upon irradiation, and that several decomposition products, including **3**, are produced (Fig 4). To further verify these conclusions, the energy of each proposed reaction pathway was calculated to confirm their validity. More specifically, the singlet–triplet (S-T) transition energy of **1** was determined to be 26.2 kcal/mol, which is higher than the excitation energy of triplet oxygen (22.6 kcal/mol) [29], thereby indicating that the photosensitization of oxygen molecules by **1** is possible. In addition, the O-O bond dissociation enthalpy of **2** was determined to be 13.7 kcal/mol. Generated enthalpies were calculated by Gaussian 16, uB3LYP/6-31+G** as function and basic. Excitation light close to 400 nm has an energy of ~70 kcal/mol, which is sufficient to cleave the O-O bond of **2**, and so these results therefore support the feasibility of the photodegradation pathway proposed in Fig 4.

## Conclusion

We successfully identified the reaction intermediates and secondary degradation products of 9,10-dibutoxyanthracene (DBA, **1**), which had not been previously elucidated. In addition, we examined the reaction conditions and proposed a reaction pathway based on the experimental evidence. Our results indicated that compound **1** was consumed upon reaction with the oxygen present in air (~21% O_2_) when irradiated with UV light, resulting in the generation and capture of singlet oxygen, in addition to the formation of 9,10-dibutoxyanthracene-endoperoxide (DBA-EPO, **2**). Compound **2** was further decomposed by light to produce secondary products such as **3**, as well as additional unidentified products. The proposed reaction pathways were supported by density functional theory calculations of the singlet-triplet transition energy of **1** and the O-O bond dissociation enthalpies of the EPO. In particular, it was demonstrated that the addition of *N*,*N*-triethylenediamine as a singlet oxygen scavenger was effective in suppressing the photodecomposition of **1**. When **1** promotes a photoreaction, it may irreversibly decrease in concentration by exciting oxygen in the system, and ultimately capturing and decomposing both singlet oxygen and itself. Compound **1** is therefore expected to promote reactions under oxygen-free conditions, such as radical reactions, as a photo-induced oxygen scavenger. For broader applications, control of the secondary decomposition of the EPO-containing compound **2** observed in this study is required.

## Supporting information

S1 File The information of equilibrium structures. (DOCX)
