# Supplementary material for: Mechanism for the photodegradation of 9,10-dibutoxyanthracene in the presence of air
Source: PLoS One. 2022 Mar 11;17(3):e0263526. doi: 10.1371/journal.pone.0263526 (PMC8916649; doi:10.1371/journal.pone.0263526)
Supplement: S1 File — (DOCX) [file pone.0263526.s001.docx]

Supplementary information on the paper

Mechanism for the photodegradation of 9,10-dibutoxyanthracene in the presence of air

Ryotaro Seto,[a] Arisa Sato,[a] Keita Iuchi,[b] Shunichi Himori,[b] Hiroaki Gotoh*[a]

[a]Department of Chemistry and Life Science

Yokohama National University

79-5 Tokiwa-Dai, Hodogaya-Ku, Yokohama 240-8501 JAPAN.

E-mail: gotoh-hiroaki-yw@ynu.ac.jp

[b]Kawasaki Kasei Chemicals Ltd., Research & Development Center, 1-2 Chidori-Cho, Kawasaki-Ku, Kawasaki 210-0865 JAPAN2

The information of equilibrium structures

The information for the calculated equilibrium structures are described. Coordinates and thermochemical values (zero-point correction, thermal correction to energy, thermal correction to enthalpy, thermal correction to Gibbs free energy, sum of electronic and zero-point energies, sum of electronic and thermal energies, sum of electronic and thermal enthalpies, and sum of electronic and thermal free energies) were calculated.


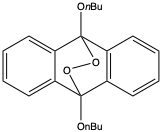


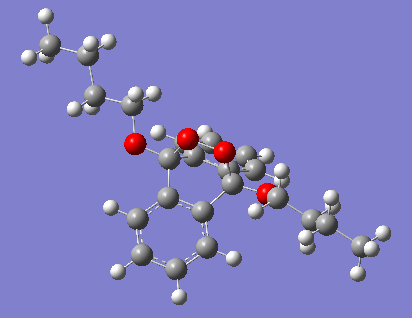


uB3LYP/6-31+G**

C -1.39042175 -1.13459755 -2.48751724

C -0.69404809 -1.24808413 -1.28335445

C 0.69406816 -1.24809905 -1.28334352

C 1.39048867 -1.13463911 -2.48748331

C 0.69691973 -1.02790341 -3.68431382

C -0.69681487 -1.02788352 -3.68432955

C -1.39047960 -1.14113382 2.48500734

C -0.69692092 -1.03756056 3.68211588

C 0.69681578 -1.03754076 3.68213168

C 1.39041241 -1.14109247 2.48504141

C 0.69404742 -1.25139112 1.28057349

C -0.69406776 -1.25140596 1.28056250

C 1.51068169 -1.40971866 -0.00156455

O 1.93906065 -2.69875281 -0.00352866

O 2.63470202 -0.55701885 -0.00048527

C -1.51068181 -1.40971853 -0.00160317

O -2.63470208 -0.55701866 -0.00052679

O -1.93906090 -2.69875298 -0.00336085

H -2.47404590 -1.12306729 -2.46442216

H 2.47411190 -1.12314634 -2.46434852

H 1.24089175 -0.94517269 -4.61750185

H -1.24076146 -0.94513557 -4.61753116

H -2.47410421 -1.12959075 2.46191251

H -1.24089586 -0.95728860 4.61551697

H 1.24076527 -0.95725165 4.61554640

H 2.47403794 -1.12951213 2.46198639

C 2.35498458 0.83705627 0.00123708

H 1.76632480 1.10558580 -0.88570832

H 1.76677483 1.10350855 0.88910218

C 3.67783313 1.57792275 0.00175863

H 4.25361068 1.26997763 0.88040854

H 4.25314320 1.27203867 -0.87791711

C 3.48807895 3.09306551 0.00358117

H 2.90156330 3.38749233 -0.87330898

H 2.90200608 3.38544178 0.88145272

C 4.81915064 3.84079979 0.00412077

H 5.40996142 3.58113950 0.88605409

H 5.40954941 3.58314601 -0.87867652

H 4.67096316 4.92216037 0.00538482

C -2.35498454 0.83705628 0.00132711

H -1.76695407 1.10344955 0.88932947

H -1.76614550 1.10564432 -0.88548076

C -3.67783292 1.57792318 0.00162703

H -4.25380223 1.26989820 0.88012293

H -4.25295117 1.27211946 -0.87820237

C -3.48807864 3.09306569 0.00362916

H -2.90219991 3.38536121 0.88165709

H -2.90136891 3.38757278 -0.87310428

C -4.81915017 3.84080039 0.00394381

H -5.40935393 3.58322884 -0.87900785

H -5.41015590 3.58105838 0.88572243

H -4.67096261 4.92216080 0.00534116

Zero-point correction= 0.436827 (Hartree/Particle)

Thermal correction to Energy= 0.461476

Thermal correction to Enthalpy= 0.462420

Thermal correction to Gibbs Free Energy= 0.381083

Sum of electronic and zero-point Energies= -1154.422201

Sum of electronic and thermal Energies= -1154.397552

Sum of electronic and thermal Enthalpies= -1154.396608

Sum of electronic and thermal Free Energies= -1154.477945


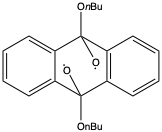


uB3LYP/6-31+G**


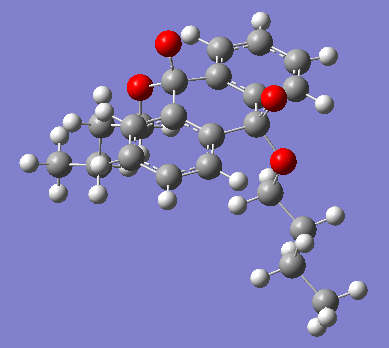


C -1.39530070 -1.18538034 -2.50386264

C -0.69947686 -1.25451389 -1.28828646

C 0.69936352 -1.25453455 -1.28834740

C 1.39515895 -1.18550709 -2.50396348

C 0.69927699 -1.11788822 -3.71018474

C -0.69947450 -1.11783059 -3.71013117

C -1.39512729 -1.18379165 2.50485025

C -0.69928308 -1.11551170 3.71103923

C 0.69947063 -1.11547048 3.71098651

C 1.39525880 -1.18369436 2.50475037

C 0.69945529 -1.25351176 1.28919393

C -0.69935232 -1.25352176 1.28925468

C 1.52047391 -1.39065584 0.00049995

O 1.95783984 -2.67756671 -0.00036782

O 2.65113019 -0.52047717 0.00015412

C -1.52047838 -1.39065273 0.00060237

O -2.65113244 -0.52047739 0.00031402

O -1.95783909 -2.67756798 0.00051074

H -2.47990351 -1.17772596 -2.48566519

H 2.47976098 -1.17796979 -2.48579967

H 1.24632875 -1.07055358 -4.64704596

H -1.24658734 -1.07045622 -4.64695569

H -2.47973663 -1.17627285 2.48672222

H -1.24634675 -1.06764127 4.64786777

H 1.24659468 -1.06757131 4.64777913

H 2.47986892 -1.17607857 2.48658914

C 2.39444666 0.88884354 -0.00006142

H 1.80890759 1.16701737 -0.88828668

H 1.80932999 1.16740098 0.88831030

C 3.73550149 1.61232653 -0.00054536

H 4.30922059 1.29345294 0.87903902

H 4.30879900 1.29308190 -0.88026984

C 3.58258489 3.13934217 -0.00082871

H 2.99949346 3.44865688 -0.87928671

H 2.99989610 3.44902295 0.87776668

C 4.92868089 3.87307005 -0.00129111

H 5.52166222 3.61230448 0.88306341

H 5.52125608 3.61193713 -0.88580976

H 4.79018313 4.95946311 -0.00148470

C -2.39444444 0.88884332 0.00007297

H -1.80942568 1.16741615 0.88850629

H -1.80880503 1.16699546 -0.88809103

C -3.73549691 1.61232981 -0.00057564

H -4.30934293 1.29341573 0.87891090

H -4.30866929 1.29312927 -0.88039797

C -3.58257536 3.13934523 -0.00076529

H -3.00005676 3.44898457 0.87795724

H -2.99931190 3.44869750 -0.87909611

C -4.92866887 3.87307722 -0.00145730

H -5.52107076 3.61198918 -0.88610524

H -5.52182516 3.61227057 0.88276779

H -4.79016760 4.95946984 -0.00157088

Zero-point correction= 0.432409 (Hartree/Particle)

Thermal correction to Energy= 0.457670

Thermal correction to Enthalpy= 0.458614

Thermal correction to Gibbs Free Energy= 0.373574

Sum of electronic and zero-point Energies= -1154.404929

Sum of electronic and thermal Energies= -1154.379668

Sum of electronic and thermal Enthalpies= -1154.378724

Sum of electronic and thermal Free Energies= -1154.463764
